# Meta-analysis of grey matter changes and their behavioral characterization in patients with alcohol use disorder

**DOI:** 10.1038/s41598-021-84804-7

**Published:** 2021-03-04

**Authors:** Carolin Spindler, Sebastian Trautmann, Nina Alexander, Sonja Bröning, Sarah Bartscher, Markus Stuppe, Markus Muehlhan

**Affiliations:** 1 Department of Psychology, Faculty of Human Sciences, MSH Medical School Hamburg, Am Kaiserkai 1, 20457 Hamburg, Germany; 2 Department of Pedagogy, Faculty of Health Sciences, MSH Medical School Hamburg, Hamburg, Germany; 3 Hafencity Institute for Psychotherapy, MSH Medical School Hamburg, Hamburg, Germany; 4 Department of Addiction Medicine, Carl-Friedrich-Flemming-Clinic, Helios Medical Center Schwerin, Schwerin, Germany

**Keywords:** Addiction, Addiction

## Abstract

Alcohol Use Disorder (AUD) is associated with reductions in grey matter (GM) volume which can lead to changes in numerous brain functions. The results of previous studies on altered GM in AUD differ considerably in the regions identified. Three meta-analyses carried out between 2014 and 2017 yielded different results. The present study includes the considerable amount of newer research and delivers a state-of-the art meta-analysis in line with recently published guidelines. Additionally, we behaviorally characterized affected regions using fMRI metadata and identified related brain networks by determining their meta-analytic connectivity patterns. Twenty-seven studies with 1,045 AUD patients and 1,054 healthy controls were included in the analysis and analyzed by means of Anatomical Likelihood Estimation (ALE). GM alterations were identified in eight clusters covering different parts of the cingulate and medial frontal gyri, paracentral lobes, left post- and precentral gyri, left anterior and right posterior insulae and left superior frontal gyrus. The behavioral characterization associated these regions with specific cognitive, emotional, somatosensory and motor functions. Moreover, the clusters represent nodes within behaviorally relevant brain networks. Our results suggest that GM reduction in AUD could disrupt network communication responsible for the neurocognitive impairments associated with high chronic alcohol consumption.

## Introduction

Alcohol use disorder (AUD) has a high worldwide prevalence and is associated with an enormous health burden, as well as increased mortality^1–3^. AUD also leads to impairments and problems at work (e.g., not meeting job requirements), elevated risk of injuries (e.g., endangerment or traffic accidents) and negative consequences in social and family contexts (e.g. neglect of social relationships, aggression toward family members)^4^.

AUD trajectories are characterized by changes in numerous cognitive and affective processes that play a vital role for disorder development, maintenance and relapse^5,6^. Continuously high levels of alcohol consumption can lead to long-term functional changes, such as impairments in visuo-spatial functioning, i.e. perceiving and remembering locations^7,8^ and in higher cognitive functioning, such as self-control, planning, inhibition, reasoning and explicit emotion regulation^9–11^. Changes in affective processes such as emotional instability^12^ and mood disturbances may also be related to heavy alcohol consumption^13^ and might explain the high co-morbidity of AUD with depressive disorders^14^. AUD-induced impairments of cognitive and affective functions are furthermore associated with poorer therapeutic outcomes^14,15^.

Among other factors, functional impairments in AUD can be explained by changes in grey matter (GM) structure, resulting from the neurotoxicity of alcohol in chronic high consumption patterns^16–18^. Numerous studies indicate widely distributed GM volume reductions in AUD patients compared to healthy controls^19–23^. The results of these individual studies refer to various cortical and subcortical regions that are altered by alcohol consumption. However, the identified brain regions vary considerably across these different studies. The inconsistency of individual neuroimaging results may be explained by study characteristics such as sample size, comorbidity, analytical and experimental variability^24^. In order to interpret the existing findings and quantitatively integrate effects across individual studies, meta-analytic approaches have been developed^24,25^. Thus far, three meta-analyses^26–28^ integrated the results of studies that analyzed GM changes in AUD patients compared to healthy controls using voxel-based morphometry (VBM^29^). Xiao et al.^26^ identified nine eligible VBM studies (published from January 2000 to November 2014) and included neuroimaging data from 269 AUD patients compared to 359 healthy controls in an effect-size based meta-analytical approach. Their resulting meta-analytic maps indicated significantly reduced GM in both hemispheres comprising parts of the prefrontal cortices (PFC), anterior cingulate cortices (ACC), striatal and insular regions and parts of the posterior cingulate cortices (PCC) in AUD patients compared to healthy controls.

Yang et al.^27^ identified twelve eligible VBM studies (published from January 2000 to December 2014). They also compared whole-brain GM differences between 433 AUD patients and 498 healthy controls with an effect-size based meta-analytical approach. In this meta-analysis, AUD patients showed decreased GM in the left and right insula, superior temporal gyrus (STG), striatum, precentral gyri, dorsal lateral prefrontal cortices (dlPFC), anterior cingulate cortices (ACC) as well as the left thalamus and right hippocampus, relative to healthy controls.

A third meta-analysis conducted by Klaming et al.^28^ examines shared GM changes across AUD and posttraumatic stress disorder. They conducted meta-analyses for each condition compared to healthy controls. Here we only report the results on the AUD vs. HC contrast. The authors identified thirteen eligible studies (published from January 2000 to December 2017) and included the data of 456 AUD patients compared to 522 healthy controls in another effect-size based meta-analytical approach. Their results indicate GM reductions in AUD patients in middle and anterior cingulate cortices, insulae and lenticular nuclei and superior frontal gyri of both hemispheres.

In sum, prior studies have shown changes in GM volume of different brain regions that may be subject of functional changes in AUD. Three meta-analyses have integrated the existing evidence from GM changes in AUD over a period from 2000 to 2017. The results from all three meta-analyses indicate GM changes located in different parts of the PFC, ACC, striatum and the insulae. However, the studies also differed markedly in their results. The findings from Xiao et al.^26^ of reduced GM in the left and right posterior cingulum were not replicated, instead, Yang et al.^27^ additionally detected GM alterations in left and right precentral gyri as well as in subcortical regions like the left thalamus and right hippocampus. Klaming et al.^28^ did not identify these subcortical regions, but additional clusters in the left and right superior frontal gyri. Those inconsistencies could be due to methodological differences (e.g. differences in inclusion criteria or liberal statistical thresholds). Furthermore, according to the current guidelines^25^, the three meta-analyses are also underpowered (insufficient number of included studies), which bears the risk that the results are driven by a few dominant study results.

The aim of our study was therefore to re-visit the area of GM changes in AUD and to calculate the convergence of the individual findings using a state-of-the-art meta-analysis. Moreover, since 2017, new studies have continued to investigate the links between AUD and changes in GM. The inclusion of a larger number of studies facilitates the detection of smaller effects, increases robustness to generalizations, and may help resolve divergent previous findings^25,30^. Activation Likelihood Estimation (ALE)^30–32^ was chosen as the method of analysis. It ensures an excellent spatial weighting of the isolated coordinates. Additionally, we extend prior findings by characterizing the resulting ALE-clusters with regard to their behavioral profile using metadata from the BrainMap database^33^. This data-driven approach provides a better understanding of behavioral profiles related to AUD. Finally, we perform a meta-analytic connectivity modelling analysis (MACM)^34,35^ using the same database to determine in which neural networks the resulting clusters could represent potential nodes. This behavioral profile and MACM approach provides a reliable basis for future functional analysis.

## Methods

Details of the protocol for this meta-analysis were registered on PROSPERO and can be accessed at https://www.crd.york.ac.uk/prospero/display_record.php?ID=CRD42020190710.

### Literature search, study selection and data extraction

The search for neuroimaging studies investigating GM differences in patients with AUD and healthy controls was conducted on PubMed, PsycINFO and Web of Science databases (up to June 1, 2020) and by reference-tracing of the retrieved articles. Keywords were: (Alcohol Dependence OR Dependence, Alcohol OR Alcohol Addiction OR Addiction, Alcohol OR Alcoholic Intoxication, Chronic OR Chronic Alcoholic Intoxication OR Intoxication, Chronic Alcoholic OR Alcohol Use Disorder OR Alcohol Use Disorders OR Use Disorder, Alcohol OR Use Disorders, Alcohol OR Alcohol Abuse OR Abuse, Alcohol) AND (voxel-based morphometry OR VBM OR structural MRI). We used the following inclusion criteria: (1) written in English language and peer-reviewed, (2) statistical comparison of GM voxel-based-morphometry data from a group of patients diagnosed with AUD (DSM-IV, DSM-5 or ICD-10) and a healthy control group. We refer to AUD (as specified in DSM-5) as a disorder continuum subsuming DSM-IV criteria of alcohol abuse and alcohol dependence as well as ICD-10 criteria of harmful use and dependence syndrome of alcohol^36,37^. (3) Results were reported as 3-D coordinates in a standard reference space (e.g. MNI or Talairach)^38,39^.

**Figure 1:**
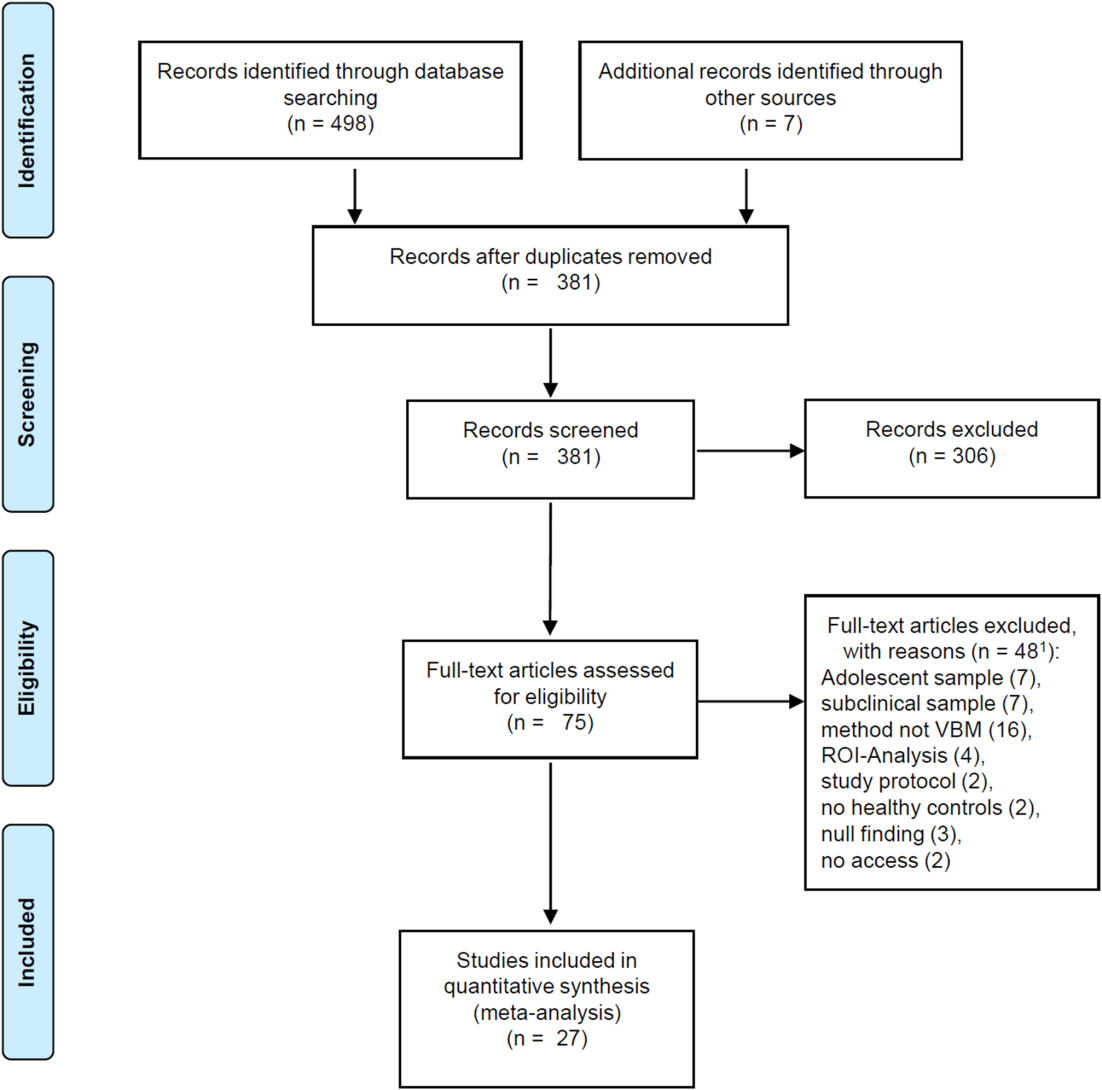
Flow diagram on the different phases of the systematic literature search. ^1^Differences in numbers of excluded articles and exclusion reasons result from studies excluded because of multiple exclusion criteria.

Exclusion criteria comprised (1) review-studies, meta-analyses and re-analyses, (2) region of interest analyses, small volume corrected results and experiments with only partial brain coverage, (3) methodological studies and study protocols, (4) studies with small sample sizes (< 10 per group) and (5) studies with statistical approaches not correcting for multiple comparisons or setting a minimum cluster extension as statistical threshold for significance^24,25,31^. Unlike conventional meta-analytical methods, (6) studies reporting null-findings could not be taken into account because they do not provide spatial coordinates, which are a prerequisite for coordinate based methods like the ALE^31^. (7) Studies investigating patient groups with Korsakow syndrome, with other primary psychopathology or comorbid other substance use disorder (except for nicotine) and studies investigating adolescent samples were also excluded. The flow of information through the different phases of the literature search is shown in Fig. 1 and is orientated on Preferred Reporting Items for Systematic Reviews and Meta-Analysis (PRISMA-Statement)^40^.

The data of the eligible studies was manually extracted (C.S.) and double-checked by a second investigator (M.M.). The extraction included demographic sample characteristics, methodological characteristics and the peak voxel coordinates of the experiments reported in the studies included in the meta-analysis. In four studies, the peak voxel coordinates were not explicitly reported. For two of these studies, the data was provided after personal correspondence with the authors^41,42^.

In order to assess risk of bias and for quality assessment, C.S. and M.M. independently checked for concordance with recently published guidelines for neuroimaging meta-analysis^25^. Except for one study, only GM decreases in AUD patients compared to healthy controls (HCs) were reported. In the study that reported both peak voxel coordinates for the contrast AUD < HCs as well as AUD > HCs^43^, we pooled the foci into one experiment for the analysis as recommended to avoid dependence across experiment masks. In eight additional studies (Supplementary table S1), we suspected a potential sample overlap even though it was not explicitly stated in the manuscript, since the studies were published from the same research group and they included patients from the same hospitals and sociodemographic and psychometric data were approximately comparable. To further minimize the risk of bias, we have also pooled their foci into one experiment and thus adopted a conservative approach by considering the smaller sample size. To take the potential of publication bias into account, conventional detection methods as for effect-size meta-analyses cannot be applied in the ALE-approach^25^. Therefore, a post-hoc noise simulation, referring to a modified version of the fails-safe N (FSN) method^44^, was applied for estimation of robustness against unpublished findings.

### Anatomical likelihood estimation

To detect above-chance convergence among the reported coordinates we conducted the meta-analysis of voxel-based morphometry imaging results by applying a technique of anatomical likelihood estimation that is derived from the principles of activation likelihood estimation^30–32^. The analysis was performed using BrainMap GingerALE v3.0.2 (http://brainmap.org). Coordinates reported in Talairach space were transformed into MNI space using the Lancaster transform icbm2tal implemented in GingerALE^45,46^.

In the first step, ALE models the input coordinates with a Gaussian function, which accommodates the spatial uncertainty of the reported coordinates (e.g., resulting from variance in neuroanatomy or caused by the use of various brain templates and normalization strategies) and takes the sample size of each experiment into account^30^. In the second step, a map of the whole brain is constructed for each experiment, assigning to each voxel a value equal to the probability that a volume difference between patients and healthy controls lies within the voxel. Across all experiments, these maps are merged and yield the ALE image with the ALE values that represent the likelihood that volume differences were found at least for one experiment at a given voxel^47^. The statistical significance of the ALE scores is tested in the third and last step. A cluster level family wise error correction (cFWE) with *p* < 0.001 was used as the cluster-forming threshold and *p* < 0.05 was used as the FWE threshold, since they entail low susceptibility to false positives in terms of convergence^25,48^. In cFWE thresholding, the experimental ALE scores are tested against a null hypothesis utilizing a permutation procedure with 1000 permutations based on the identical number of foci, subjects, and experiments to generate random datasets. Where appropriate, specific subgroups will be investigated by additional analyses.

### Analysis of behavioral domain profiles and paradigm analysis

In order to assess mental processes that can be associated with the identified ALE clusters, a behavioral profile was generated using the “Behavioral Analysis Plugin Version 3.1” for Mango 4.1 (http://rii.uthscsa.edu/mango/). This plugin automatically compared the identified ALE clusters with functional metadata of the BrainMap Database (http://brainmap.org). BrainMap’s behavioral domains are Action, Cognition, Emotion, Interoception and Perception, subdivided into sixty sub-domains. At that time (July 8^th^, 2020), the BrainMap functional database contained 3.628 papers with 18,079 experiments. Before the behavioral analysis was conducted, the ALE clusters were stored as binary masks and transformed into the Talairach space using the MNI-to-Tal transform option in Mango (http://ric.uthscsa.edu/mango/). The comparison of the proportions of behavioral domains found within the selected cluster with the proportions of the behavioral domains across the whole BrainMap database results in a behavioral domain profile of the selected clusters. *Z*-Scores ≥ 3.0 with *p* ≤ 0.05 (corrected for multiple comparisons) are considered significant^33^. In addition to the behavioral analysis we also conducted a regional paradigm analysis using the “Paradigm Analysis Plugin Version 1.6” for Mango v4.1. Here, the results of the comparison of our ALE clusters and the literature in the BrainMap Database are presented for 111 paradigm classes that also characterize functional metadata. For paradigm analysis, the significance threshold is set at *Z*-Scores ≥ 3.3 with *p* ≤ 0.05 (corrected for multiple comparisons; http://ric.uthscsa.edu/mango/versionhistory.html#v401).

### Meta-analytic connectivity modeling

To examine the functional networks of the ALE-derived clusters we used a region-to-whole-brain meta-analytic connectivity modeling approach (MACM)^34,35^. Such analysis is based on the co-occurrence of spatially separated neurophysiological events and – when used in conjunction with the BrainMap database – results in a large-scale analysis that includes decades of neuroimaging data. Thus, MACM is a data-driven method, performed using BrainMap Sleuth v3.0.4 and BrainMap GingerALE v3.0.2 (http://brainmap.org) that is useful for identifying connections within an indirect network.

First, we acquired anatomical seed regions of interest (ROIs) of our ALE-clusters using Mango v4.1. Next, we transformed and stored them as Talairach images as required for Sleuth’s image search capability. We conducted our search using Sleuth with the anatomical seed ROIs and the following additional search criteria: “Diagnosis: Normals”, “Context: Normal Mapping”, “Imaging Modality = fMRI OR PET” and “Activations: Activations Only”. The identified co-activation coordinates were exported as input data for GingerALE. We then conducted a quantitative meta-analysis applying the ALE-algorithm as described previously (corrected for multiple comparison with cFWE-corrected threshold with *p* < 0.05), resulting in areas of convergence among the co-activation coordinates^49^.

## Results

**Figure 2:**
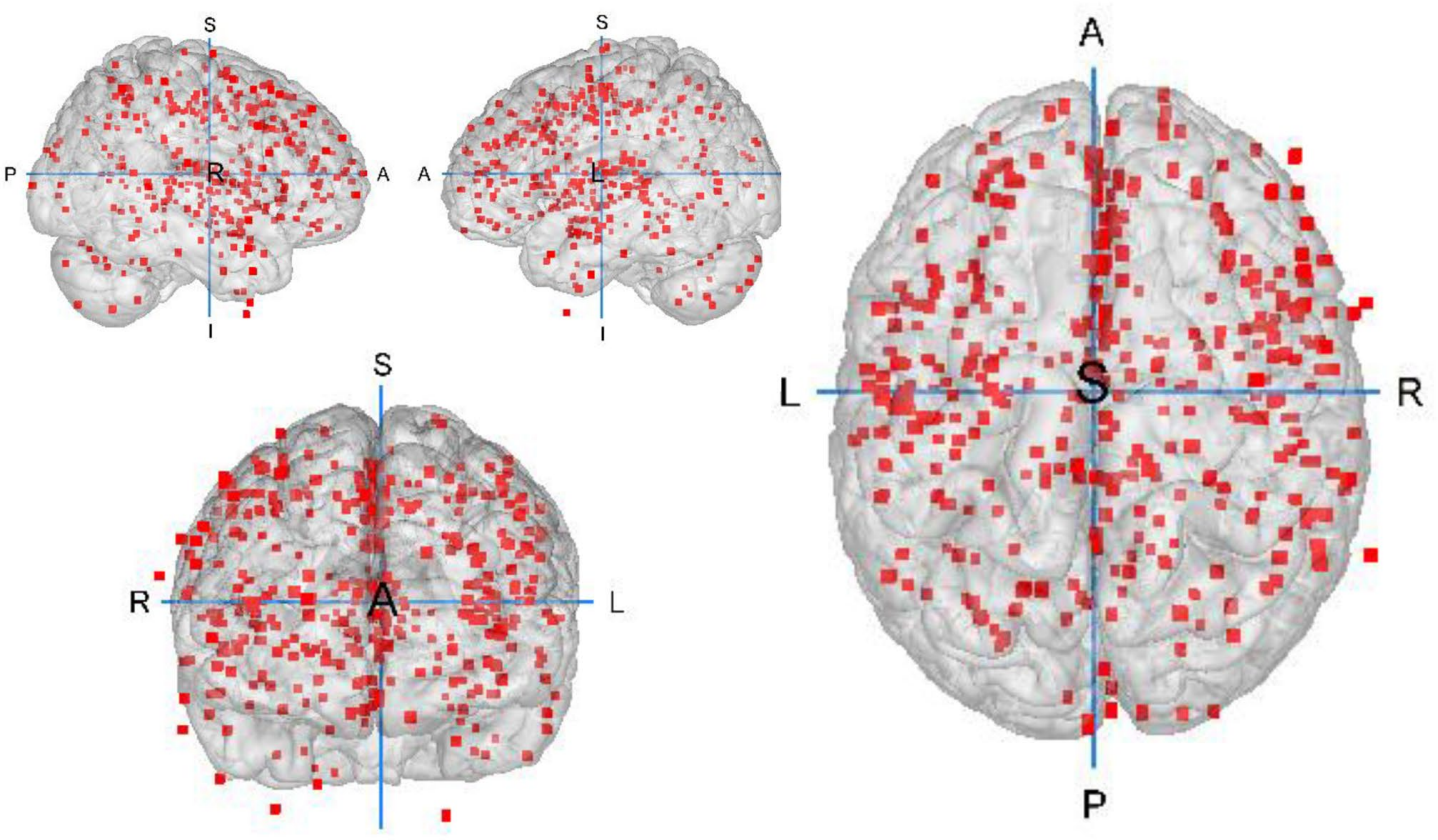
Foci-distribution of the experiments included in the ALE meta-analysis. Foci are depicted on a grey matter glass brain. Red Blobs: Foci; A: anterior; S: superior; P: posterior; L: left; R: right. This image was created with Mango v4.1. (http://ric.uthscsa.edu/mango/).

Twenty-seven studies were eligible and therefore included in the meta-analysis. These studies entail a total 1,045 AUD patients and 1,054 HCs. After pooling the foci as described in the methods section, the ALE was calculated over 23 experiments with 376 reported peak voxel coordinates^19–23,41–43,50–68^. Demographic and clinical sample characteristics of the studies included are presented in Table 1 and Fig. 2 displays the foci-distribution of the included experiments. Further information about data acquisition and data analysis of the individual studies can be found in Supplementary Table S2.

**Table 1:** Demographic and clinical sample characteristics of the studies included in the ALE meta-analysis.

#	Source	AUD patients			Duration of AUD in years *M* (*SD*)	Duration of abstinence d/w/mo, *M* (*SD*)	Healthy controls	
		n (Fem.)	Age, *M* (*SD*)	Diagnosis (diagnosis criteria)			n (Fem.)	Age, *M* (*SD*)
1	Asensio et al.^66^	24 (0)	35.62 (4.81)	Alc. Abuse (DSM-IV)	4.71 (2.93)	40.88 d (29.07)	24 (0)	31.91 (9.34)
2	Bach et al.^67^	74 (19)	46.5 (10.0)	Alc. Dependence (DSM)	13.6 (10.0)	n.a.	43 (16)	46.5 (9.2)
3	Bach et al.^50^	62 (14)	47.6 (9.7)	Alc. Dependence (DSM-IV)	n.a.	11.77 d (9.03)	74 (16)	44.12 (9.6)
4	Chanraud et al.^19^	26 (0)	47.7 (7.1)	Alc. Dependence (DSM-IV)	8 (6.3)	26.4 w (29.0)	24 (0)	45.0 (6.72)
5	Chanraud et al.^51^	24 (0)	47.8 (7.7)	Alc. Dependence (DSM-IV)	9.2 (8.9)	31.0 w (31.0)	24 (0)	45.0 (5.6)
6	Charlet et al.^43^	40 (10)	44.9 (11.4)	Alc. Dependence (DSM-IV)	8.95 (9.1)	11.95 d (5.55)	40 (10)	44.1 (12.0)
7	Demirakca et al.^20^	50 (23)	46.6 (8.2)	Alc. Dependence (DSM-IV, ICD-10)	12.4 (7.4)	16.5 d (7.3)	66 (32)	45.0 (10.1)
8	Galandra et al.^52,85^	23 (9)	45.69 (7.82)	Alc. Dependence (DSM-V)	10.8 (7.21)	≥ 10 d	18 (7)	44.83 (8.86)
9	Galandra et al.^53^	22 (9)	45.59 (7.99)	Alc. Dependence (DSM-IV)	11.89 (7.11)	≥ 10 d	18 (8)	44.83 (8.86)
10	Grodin et al.^21^	37 (16)	40.2 (9.2)	Alc. Dependence (DSM-IV)	10.3 (7.5)	21.5 d (5.3)	69 (22)	36.6 (1.1)
11	Guggenmos et al.^54^	119 (18)	45.0 (10.7)	Alc. Dependence (DSM-IV, ICD-10)	11.7 (9.9)	22.8 d (11.5)	97 (16)	43.7 (10.8)
12	Jang et al.^55^	20 (0)	43.5 (6.0)	Alc. Dependence (DSM-IV)	n.a.	7.8 d (6.5)	20 (0)	44.5 (7.4)
13	Mechtcheriakov et al.^22^	22 (8)	53.6 (n.a.)	Alc. Addiction (ICD-10)	n.a.	n.a.	22 (8)	53.7 (n.a.)
14	Nurmedov et al.^68^	24 (4)	40.79(9.807)	Alc. Use Disorder (DSM5)	7.7 (4.75)	≥ 3 d	29 (6)	37.45 (10.871)
15	Pitel et al.^42^	34 (6)	43.47 (8.36)	Alc. Dependence (DSM-IV)	16.09 (10.29)	12.67 d (6.94)	25 (14)	43.88 (11.24)
16	Rando et al.^56^	45 (10)	38.20 (7.74)	Alc. Dependence (DSM-IV)	n.a.	35.12 d (7.3)	50 (22)	31.14 (9.04)
17	Reiter et al.^57^	43 (9)	44.42(10.21)	Alc. Dependence (DSM-V, ICD-10)	14.64 (9.96)	28.8 d (11.85)	35 (10)	42.0 (10.49)
18	Ritz et al.^41^	17 (4)	44.35 (9.17)	Alc. Dependence (DSM-IV)	9.17 (7.14)	16.25 d(23.29)	16 (4)	45.44 (7.14)
19	Rosenthal et al.^65^	46 (16)	44.56(11.18)	Alc. Dependence (DSM-IV)	n.a.	n.a.	39 (23)	42.74(11.69)
20	Segobin et al.^58^	19 (2)	44.40 (6.07)	Alc. Dependence (DSM-IV)	8.22 (8.79)	11.05 (5.20)	20 (n.a.)	46.7 (4.25)
21	Trick et al.^59^	29 (11)	40.4 (9.55)	Alc. Dependence (DSM-IV)	n.a.	≥ 2 w	31 (15)	40.2 (8.7)
22	Van Eijk et al.^60^	49 (9)	47.0 (10.1)	Alc. Dependence (DSM-IV, ICD-10)	n.a.	1 d	55 (13)	45.3 (11.9)
23	van Holst et al.^23^	36 (0)	43.2 (11.03)	Alc. Abuse or Dependence (DSM-IV)	11.69 (9.7)	18 d (n.a.)	54 (0)	35.3 (10.1)
24	Wang et al.^61^	20 (0)	43.95 (6.30)	Alc. Dependence (DSM-IV)	n.a.	41.5 d (10.80)	20 (0)	40.50 (8.17)
25	Dong et al.^62^	56 (0)	43.36 (8.6)	Alc. Dependence (DSM-IV)	n.a.	50.58 d (n.a.)	33 (0)	42.88 (6.05)
26	Wiers et al.^63^	22 (0)	42.14 (6.2)	Alc. Dependence (DSM-IV)	14.82 (7.4)	48.32 d (46.95)	21 (0)	41.95 (6.41)
27	Zois et al.^64^	95 (24)	45.9 (9.9)	Alc. Dependence (DSM-IV)	10.9 (8.9)	11.69 d (6.66)	87 (16)	45.9 (10.6)

AUD = alcohol use disorder, Fem. = females, d/w/mo = days/weeks/months, n.a. = information not available.

### Significant ALE clusters

**Figure 3:**
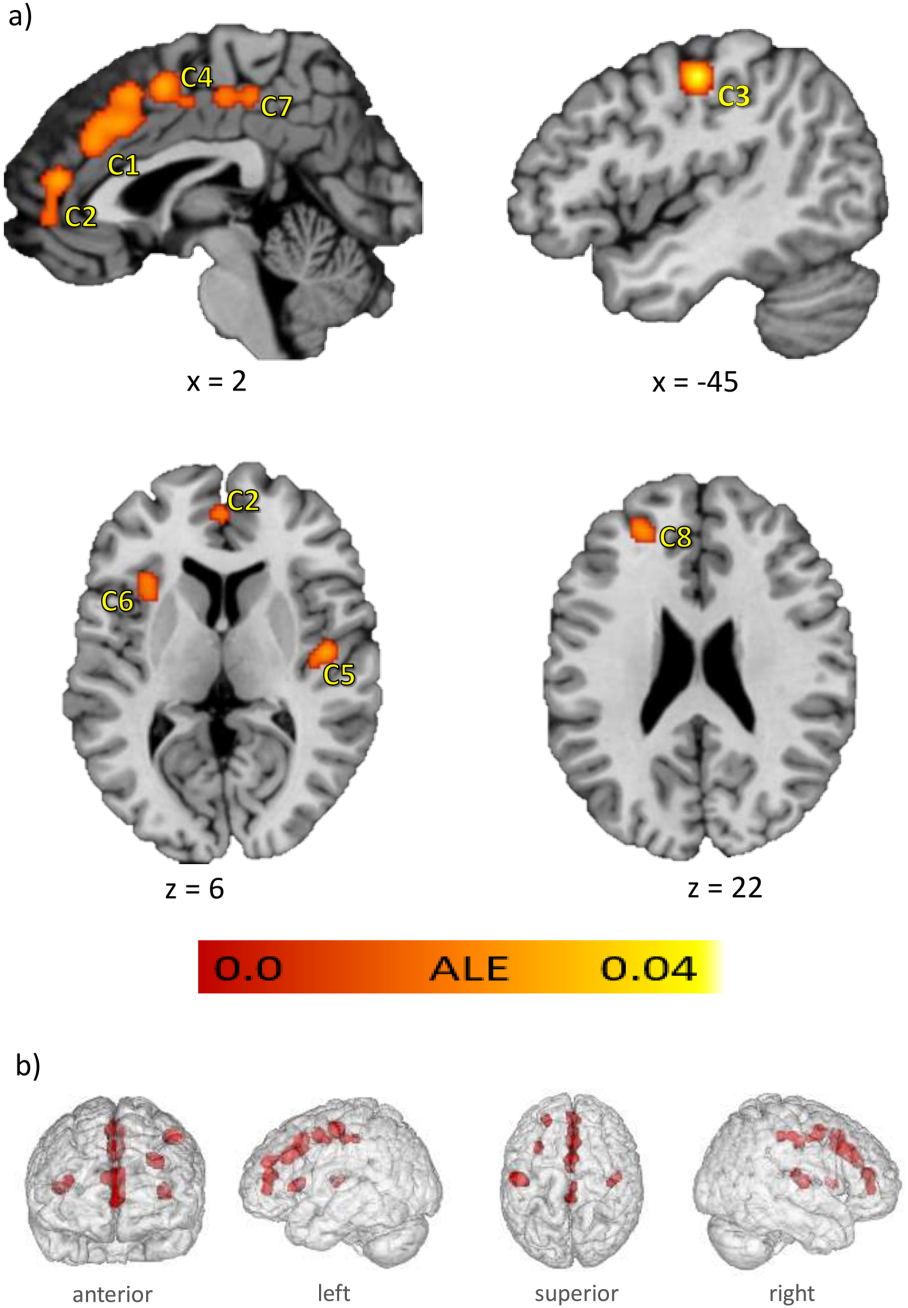
Results of the ALE meta-analysis. The highlighted clusters (C1–C8) represent significant convergence about grey matter differences in AUD patients compared to healthy controls. (**a**) Clusters are depicted on brain slices of an MNI standard brain. Color indicates ALE value. (**b**) Spatial position and expansion of the ALE-clusters depicted on a grey matter glass brain. Cluster-forming threshold *p* < 0.001, family wise error (FWE) cluster level corrected at *p* < 0.05. x, y and z values refer to coordinates in MNI space, for detailed MNI peak voxel coordinates of the ALE clusters see Table 2. This image was created with Mango v4.1. (http://ric.uthscsa.edu/mango/).

The ALE meta-analysis yielded eight significant clusters of convergence, named C1 to C8 according to their spatial extent (Fig. 3a,b), with C1 being the largest cluster. Four medial clusters (C1, C2, C4 and C7) cover parts of the anterior, middle and posterior cingulate gyri (58.3% of C1, 40.0% of C4 and 25.0% of C7), the medial frontal gyri (20.0% of C1, 39.3% of C2 and 55.0% of C4), the superior frontal gyri (21.7% of C1), the paracentral lobe (5.0% of C4 and 64.3% of C7), the anterior cingulate cortices (60.7% of C2) and the precuneus (10.7% of C7) in both hemispheres. The third cluster (C3) comprises the left postcentral and precentral gyri (63.5% and 36.5%). The right posterior insula and claustrum are covered by C5 (88.6% insula and 11.4% claustrum) and left anterior insula and claustrum are covered by C6 (51.6% insula, 48.4% claustrum). The last cluster, C8, is located in the left hemisphere covering the superior frontal gyrus (42.9%), middle frontal gyrus (28.5%) and medial frontal gyrus (28.6%). The cluster sizes, the peak voxel coordinates and their ALE values, the center of mass as well as the number of contributing experiments can be found in Table 2. In general, ALE does not allow any statement about the direction of the effects. However, all included studies reported a substance reduction and only one study reported an additional volume increase in AUD in the left cuneus^43^. This region did not contribute to the identified ALE clusters. Thus, the clusters of this ALE analysis are considered as regions with reduced GM volume. Overall, 20 of the 23 included studies contributed to the ALE clusters. A detailed overview of the contributing studies per cluster is shown in Supplementary table S3.

**Table 2:** ALE clusters significant after cluster-level FWE correction for multiple comparisons.

Cluster #	Anatomical label^a^	Peak voxel coordinates (MNI)			BA	ALE (*10^–2^)^b^	Cluster Size (mm^3^)	Center of mass (x, y, z)	No. of contributing experiments (%)
		x	y	z					
1	L Cerebrum.Frontal Lobe.Cingulate Gyrus	0	20	36	32	2.72	3040	1.7,23.4,37.6	12 (52.2)
	R Cerebrum.Frontal Lobe.Superior Frontal Gyrus	4	18	48	6	2.57			
	L Cerebrum.Limbic Lobe.Cingulate Gyrus	2	32	30	32	2.37			
2	L Cerebrum.Limbic Lobe.Anterior Cingulate	0	46	14	32	2.76	1664	0.8,47.5,9.4	9 (39.1)
	L Cerebrum.Limbic Lobe.Anterior Cingulate	− 2	50	2	32	1.98			
3	L Cerebrum.Parietal Lobe.Postcentral Gyrus	− 44	− 16	48	3	4.01	1456	− 45.1, − 15.1,47.2	7 (30.4)
4	L Cerebrum.Frontal Lobe.Medial Frontal Gyrus	2	4	52	6	2.67	1264	0.2,1.2,49.3	7 (30.4)
	L Cerebrum.Frontal Lobe.Paracentral Lobule	0	− 6	46	31	1.96			
5	R Cerebrum.Sub-lobar.Insula	46	− 12	6	13	2.57	984	43.5, − 14.7,8.1	6 (26.1)
	R Cerebrum.Sub-lobar.Insula	40	− 18	12	13	2.27			
6	L Cerebrum.Sub-lobar.Claustrum	− 32	20	2		2.42	744	− 33,18.1,4	4 (17.4)
7	L Cerebrum.Limbic Lobe.Cingulate Gyrus	0	− 32	46	31	2.12	696	0.9, − 27.2,46.8	5 (21.7)
	L Cerebrum.Frontal Lobe.Paracentral Lobule	2	− 22	48	31	1.98			
8	L Cerebrum.Frontal Lobe.Superior Frontal Gyrus	− 24	44	24	9	2.51	680	− 23.7,42.6,24.1	4 (17.4)

BA, brodman area; L, left hemisphere; R, right hemisphere; x, y, z coordinates provided in MNI space.

^a^Anatomical labelling according to Talairach Daemon (nearest gray matter within 5 mm, talairach.org) associated with the peak coordinates after icbm2tal transformation.

^b^Maximum ALE value observed in the cluster.

Because a substantial proportion of the studies examined the volume of GM (as opposed to density), we additionally tested for convergence of results from only these "volume" studies (Supplementary figure S1 and table S4). The Results show five clusters that largely converge with those of the main analysis (C1, C2, C3, C4 and C8). In contrast to the main analysis, there was no convergence in posterior cingulate gyri and insular regions.

Retesting the resulting ALE-clusters with additional noise-studies in order to quantify the robustness against unpublished findings, the ALE-clusters remained significant from 13% up to 282% added noise. A detailed presentation of the FSN per cluster can be found in Supplementary table S5.

### Behavioral characterization and paradigm analysis

**Figure 4:**
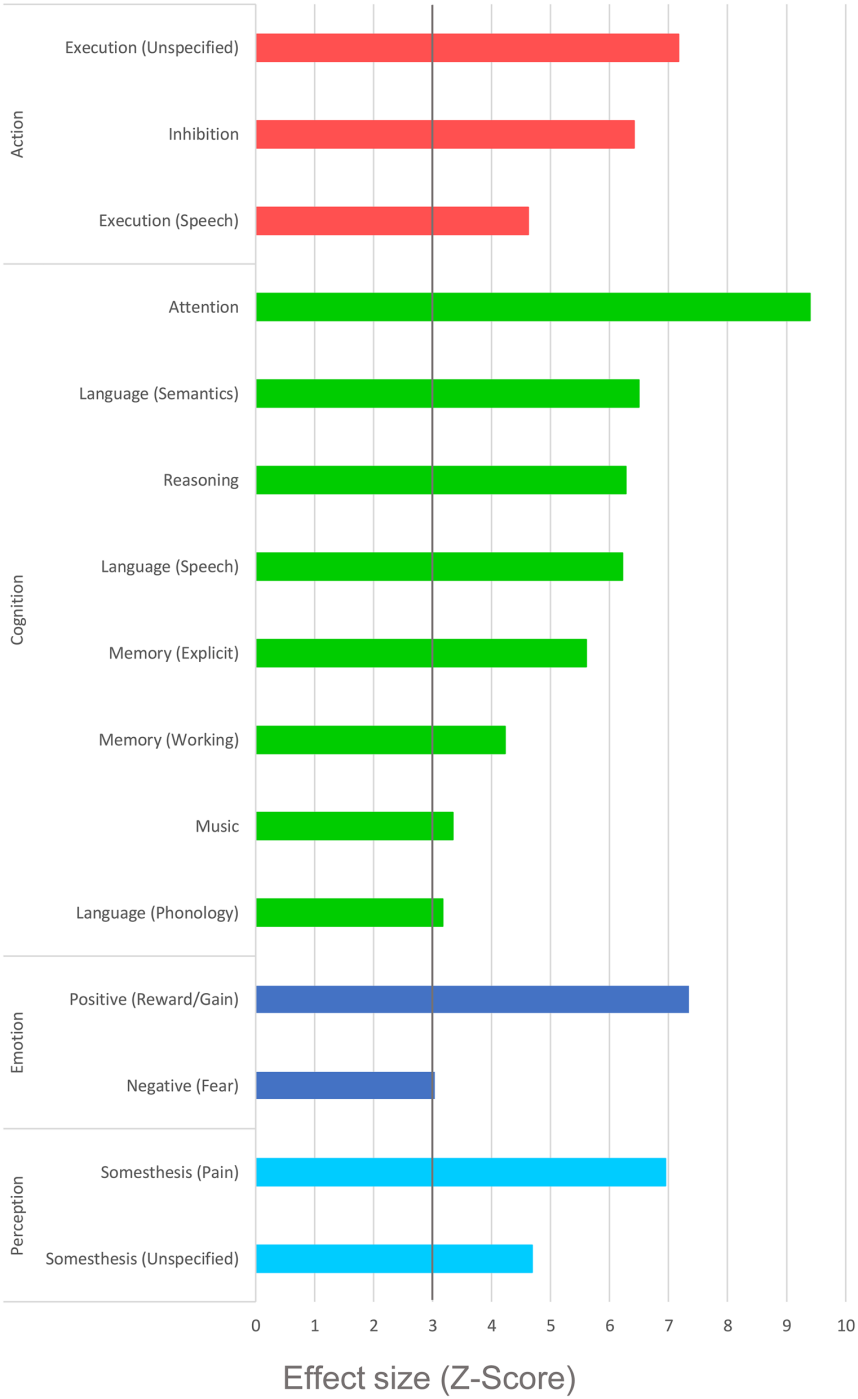
Behavioral domain profile of the ALE derived cluster-network. Only domains with an effect size of *Z* > 3 are shown, as this is significant after correction for the size of the ROI/mask and the number of domains.

**Figure 5:**
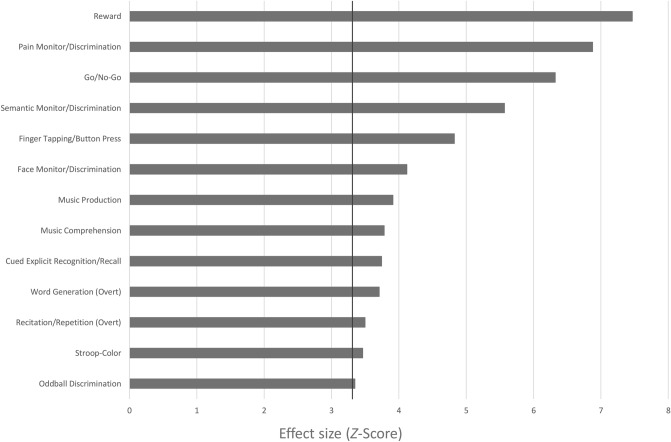
Paradigm Analysis of the ALE derived cluster-network. Only domains with an effect size of *Z* > 3.3 are shown, as this is significant after correction for the size of the ROI/mask and the number of domains.

The behavioral domain analysis of all clusters indicated that the identified pattern of structural changes is associated with several cognitive subdomains (attention, language, memory, music and reasoning), action subdomains (inhibition and execution), positive and negative emotion subdomains and perception subdomains of somesthesis (Fig. 4). The paradigm analysis revealed significant associations for thirteen paradigm classes as depicted in Fig. 5. Separate behavioral domain and paradigm analyses for each cluster are shown in Supplementary Table S6.

### Meta-analytic connectivity modelling (MACM)-analysis

**Figure 6:**
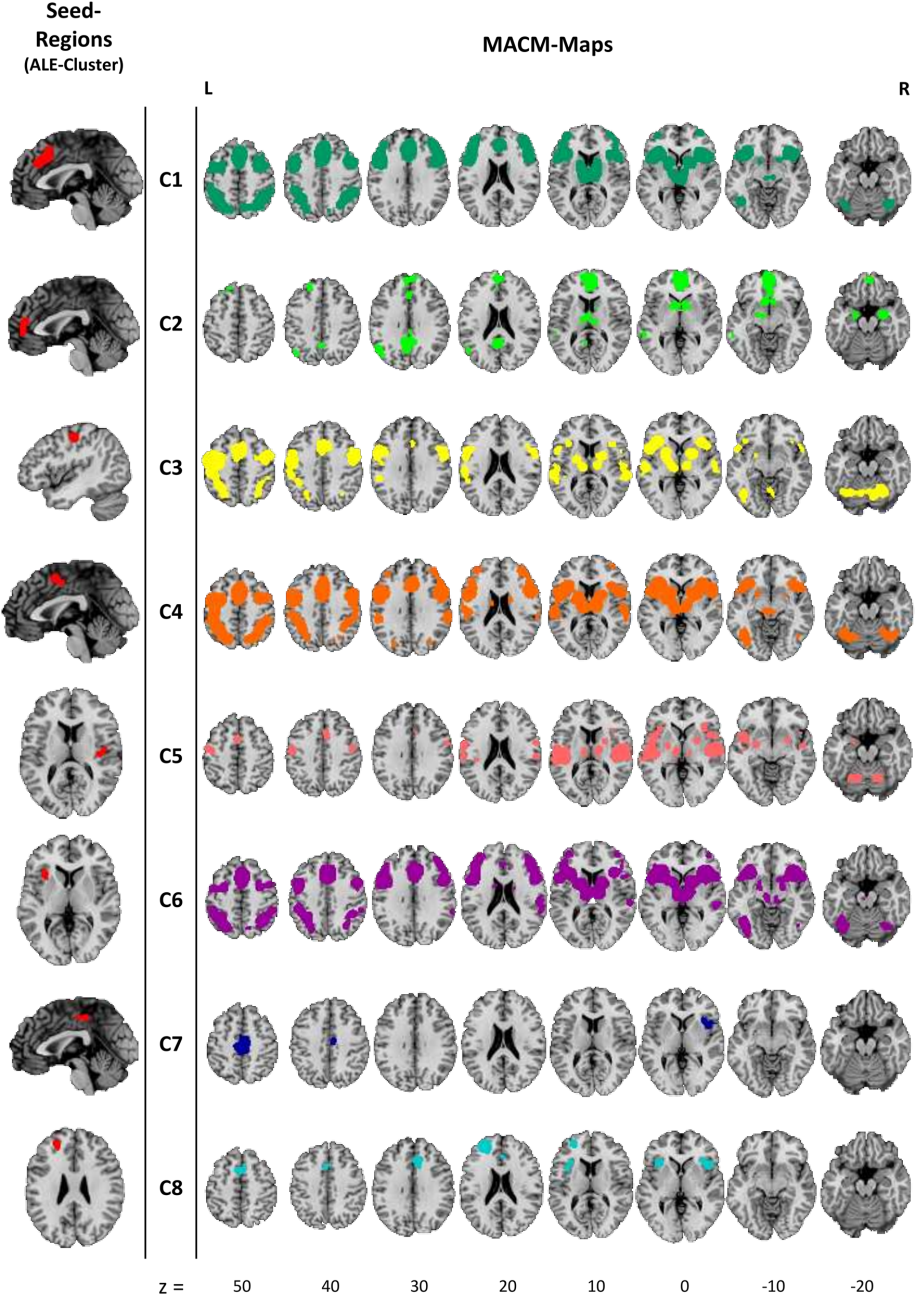
Results from the meta-analytic connectivity modelling (MACM). Left: Seed regions (ALE cluster), right: MACM-maps. All clusters were significant at a cluster-forming threshold of *p* < 0.001 and FWE cluster level corrected at *p* < 0.05. Z values indicate axial slice position in Talairach space. This image was created with Mango v4.1. (http://ric.uthscsa.edu/mango/).

The MACM-maps for C1, C3, C4 and C6 show patterns of convergent co-activation in both hemispheres for frontal gyri (middle, inferior, medial and superior), cingulate gyri, precentral gyri, parietal lobules (inferior and superior), precuneus, insulae (mid and anterior), claustrum, thalami and lentiform nuclei, postcentral and temporal co-activation patterns can be observed here too. Besides these similarities, the MACM analysis for C1 revealed convergent co-activation for anterior cingulate as well as supramarginal gyri and C1, C4 and C6 show a dorsolateral-prefrontal extension of co-activation patterns. Another similar co-activation pattern of MACM-maps for C1 and C3-C6 can be found in regions of the left and right cerebellum. The co-activation patterns for C2 also encompass frontal-, limbic- and sub-lobar regions like medial- and superior frontal gyri and the anterior cingulate as well as thalami and lentiform nuclei, precuneus, posterior cingulate and cingulate gyrus. Additionally, the left (middle) temporal lobe and left and right amygdalae are covered here too. For C5, significant co-activation was mainly observed in superior temporal gyri and mid and posterior insulae, left and right thalami, lentiform nuclei and cingulate gyri as well as precentral and postcentral, medial, and superior frontal gyri. MACM-analysis for C7 revealed a convergent co-activation pattern in both hemispheres in paracentral lobes, medial frontal, and cingulate gyri, precuneus and right anterior insula, precentral gyrus and claustrum. The co-activation patterns of C8 encompasses superior and medial frontal gyri, cingulate gyri and the anterior cingulate in both hemispheres and left and right anterior insulae, inferior frontal gyri and claustrum. All MACM-Maps are shown in Fig. 6. Further information on peak-voxel coordinates of the co-activation patterns are given in Supplementary Tables S7a–S7h.

## Discussion

By integrating the results of various studies on grey matter differences in AUD patients compared to healthy controls in a coordinate-based, meta-analytic approach, we identified eight significant clusters of convergence. These clusters indicate GM reduction in medial cortical regions such as anterior, middle and posterior cingulate gyri, frontal and superior frontal gyri, paracentral lobes and precuneus in both hemispheres. Additional clusters refer to GM reductions in left postcentral and precentral gyri, the left anterior and right posterior insulae and left superior frontal gyrus. The behavioral characterization of these clusters mainly revealed cognitive subdomains but also associations with subdomains of action, perception, and emotion. The results were further specified by analyzing the underlying paradigms, where a total of thirteen different paradigm classes with the highest effect size on reward-tasks were associated with our ALE clusters. A subsequent MACM analysis demonstrated a high degree of functional overlap within most of the clusters in lateral frontal and parietal as well as medial regions, in thalamic and insular regions. C2 additionally showed meta-analytic connectivity with left and right amygdalae and C1, C3-C6 were also linked to activation patterns in the left and right cerebellum.

The identified clusters are partially in line with the results from previous meta-analyses. In addition to the existing meta-analytical evidence, we identified clusters covering parts of the left and right paracentral lobes and the left superior frontal gyrus. Our analysis, however, did not replicate previous findings that include the right superior frontal gyrus or subcortical limbic regions like the right hippocampus, the left thalamus or the right lenticular nuclei and putamen. For prefrontal and insular regions, our results show more differentiated and less extensive clusters of convergence than previous meta-analyses. Several reasons might account for these differences. First, the applied methodology: Xiao et al.^26^, Yang et al.^27^ and Klaming et al.^28^ used effect size based methods (ES-SDM and AES-DM, e.g.^69^), which makes their strategy different from to the ALE analysis used here. While ES-SDM and AES-DM combine peak voxel coordinates with effect sizes or use statistically parametric maps of the original data, ALE weights the local maxima with the sample size^70^. Also, previous meta-analyses used more liberal statistical thresholds. Second, the number of included studies: our significantly larger number of studies compared to previous meta-analyses ensures that the findings are not skewed by a few very dominant study results^25^. The number of studies in our analysis exceeds the minimum of 17 recommended in recently published good practice guidelines by Müller et al.^25^. Third, the choice of studies: we excluded studies with adolescent samples^71–73^ because of the dynamic changes in brain anatomy throughout adolescence (e.g.^74^). We further excluded a study with a subclinical sample^75^ that was included in previous analyses. We had no access to the results of a study that was published in Chinese^76^ (see Table S8).

In sum, the findings of earlier meta-analyses were partially replicated. Diverging findings are probably due to the choice of analysis method and statistical inference approaches as well as number and type of studies included. Although different methodological approaches identified clusters in some different brain regions, our MACM analysis suggests that the resulting clusters of previous meta-analyses and the clusters of convergence in the present meta-analysis are key nodes in the same brain networks as discussed below.

While the main study question was quite broadly defined and we clarified which regions show a change in GM in AUD, a post-hoc sub-analysis was performed to investigate which regions show a change in GM volume, since most of the included experiments focus on this particular type of modulation of VBM data. The resulting ALE clusters overlap with 5/8 of our main findings. The lack of convergence in regions of posterior cingulate gyri and insular regions can be explained by the contribution of experiments that examined density rather than volume in the main analysis^22,52,53^. Thus, the methodological difference of modulated and non-modulated VBM data should be taken into account in the interpretation of the results^77^.

In order to functionally characterize the ALE derived cluster-network, we generated a behavioral domain profile in comparison with meta-data from the BrainMap database. This data driven approach allows an interpretation unbiased by presumptions and prevents effects of reverse inference^33^. The most strongly represented domain is that of cognition with subdomains indicating that the reduction of GM in AUD may be associated with changes in attention, language, memory, reasoning and music cognition. The findings are in line with a large number of studies showing changed attentional processes in AUD-patients that may be responsible for frequently reported attentional bias to alcohol-related stimuli^78,79^ or impairments of selective or divided attention, especially when semantic information is processed^80^. Furthermore, it can be assumed that impairments of semantic speech understanding, working memory and explicit memory functions lead to numerous deficits because current (task) requirements are not adequately understood, or stored, or because language responses are given incorrectly. The identified regions are also associated with higher cognitive functions, such as the formation of conclusions or judgments, that are subsumed in the reasoning subdomain. Deficits in this cognitive domain might contribute to a lack of insight into harmful alcohol consumption or difficulties in maintaining abstinence.

The results also revealed an association between positive reward processing and the ALE clusters. These findings fit well with the assumption that AUD is associated with decreased reward sensitivity that deteriorates further in the course of the disorder^81^. Reward processing and motor inhibition are also parts of the multidimensional construct of impulsivity which represents an important personality trait associated with psychopathology and the development and course of AUD (e.g.^61,82–84^). Furthermore, reduced cognitive control in combination with altered reward processing could lead to habitual behavior rather than goal-directed behavior, resulting, for example, in quick relapses into old consumption patterns for patients^85^.

The perception domain indicated that the identified regions are also related to the sensory systems associated with the skin (somesthesis), including pain perception. The paradigm analyses showed that this result was mainly driven by pain-monitor/discrimination tasks. This area of research seems to be underrepresented in alcohol use disorders^86^. The literature in the field of pain and alcohol use comprises considerations on bi-directional relations^87^. For instance, Egli et al.^88^ discuss the contribution of pain sensitivity to alcohol misuse and addiction. They argue that affective and sensory dimensions of pain in abstinence stages contribute to alcohol misuse. This might be caused due to an intersection of neural substrates that mediate nociception as well as alcohol dependence. They further argue that protracted abstinence in AUD could exacerbate dysregulated nociception. Possibly impaired somesthetic discrimination abilities, as identified in the current analysis, might contribute to this phenomenon.

Further subdomains include motor and emotional aspects. The execution of language and other non-linguistic movements as well as the inhibition of movements could be assigned to the identified clusters. Restrictions in these functions, like fine motor skills or postural abilities, are among the core symptoms of the AUD and have been repeatedly reported (for review^89^). The subdomain Fear also showed a significant effect. This finding fits well with reports of increased sensitivity to threatening situations in AUD. The enhanced sensitivity of fear- and stress-associated systems can promote alcohol consumption and continues to increase in the course of the disorder^90^.

In contrast to the previous domains, music cognition is rather difficult to assign in the existing literature. The conducted paradigm analysis explicitly revealed “music production” and “music comprehension”. While the first might be explained not only by deficits in cognitive processes but also by poor motor control (action: execution), it remains unclear to what extent deficits in music comprehension could be a burden for patients with AUD.

For a detailed description of all subdomains and paradigms see: http://brainmap.org/taxonomy/paradigms.html.

The MACM maps from the identified ALE-clusters overlap with well described nodes of several behaviorally relevant brain networks. Key nodes of the Salience Network (SN; dACC and Insulae)^91^ were covered by C1, C3-C6 and C8. Since the metadata is based on task activations, SN co-activation could be expected^92^. However, there is evidence that GM reductions in the SN have been associated with impairments of cognitive control and decision making in AUD patients^52^. In addition, the MACM-Maps of C1, C3-C6 revealed connectivity patterns in cerebellar regions, which supports the findings of previous studies on impairments of cortico-cerebellar networks in AUD that may affect motor and working memory functions^93^. Other MACM-clusters within dorsolateral prefrontal and posterior parietal regions correspond to nodes of the attention and the control network (CN) as defined by Uddin et al.^94^. Changes in these networks are likely to be responsible for the majority of impairments in higher cognitive and affective functions (e.g. updating, problem solving or response inhibition) in AUD (see^95^ for a comprehensive review). Cluster 8, in the left inferior frontal cortex, can also be counted to the Control Network and has been associated with working memory and speech processing (e. g.^96^). The posterior insulae are covered by the MACM maps that correspond to cluster 5. The posterior insular regions have been associated with interoception and pain processing^97^. GM reduction in these regions supports the findings from reduced functional connectivity^98^ and may be responsible for altered interoceptive awareness in AUD and increased sensitivity to the effects of alcohol^99,100^. Nodes of the Default Network^94,101^ are covered by the MACM maps of C2. Changes in this network can lead to disturbances of self-referential control and social interaction^95,102^. Furthermore, C2 MACM-analysis additionally shows co-activation in a fronto-striatal network (striatal, amygdalar and thalamic regions), indicating reward associated processes like reinforcement learning of craving, which play a key role in the development and maintenance of AUD^85^. The connectivity maps of clusters C3 and C7 include regions related to visuo-spatial functions and motor control^103^. Functional changes in these networks may be responsible for impairments in motor functions such as visuo-motor tracking or gait stability^89,95^.

Since the Behavioral Domain Analyses and MACM Analyses are based on the same meta-data; a further interpretation of potential dysregulated functions would be partially redundant. However, the data gives an indication in which functional brain networks the identified GM regions represent potential nodes. This offers important starting points for future research in the field of AUD. In particular, the analysis of network interactions could significantly contribute to the understanding of AUD^104^.

Besides the previously described extensions and methodological strengths of our results, some limitations need to be addressed. First, the studies included in our meta-analysis varied in terms of sample characteristics e.g. sex distribution or duration of abstinence (Table 1), which itself is reported to contribute to neuro-regeneration^18,60^. Unfortunately, it is not possible to verify the effect of these potential confounding variables using meta-regressions in ALE. If sufficient individual studies with comparable sample characteristics are available, the confounding variables should be specifically investigated by means of sub-analyses. Further heterogeneities can be seen in methodological features of the included single-studies (suppl. Table S2). Differences in inference procedures for example might have led to false positive results. These in turn can be counteracted by applying more conservative correction methods, as we did in our meta-analysis, since the cFWE-correction entails low susceptibility to false positives in terms of convergence^25,48^. Second, as already mentioned in the methods section, a limitation of the coordinate-based algorithm we used lies in possibly unnoticed publication-bias, because of its insensitivity to non-significant results^25^. However, the calculation of the FSN showed stable effects against additional noise studies. The high number of studies contributing to the ALE clusters and an FSN below the upper boundary (M = 230) also indicates that the results are not driven by a few very dominant studies^44^. Thus, a sufficient robustness to publication bias can be assumed. Third, regarding the performed functional characterization and connectivity-analysis of our results it must be considered that these results only refer to the data provided in the BrainMap database. GM changes in the identified regions are not necessarily associated with impairments in these behavioral domains and brain networks; they might also lead to improvements in the sense of a compensation mechanism. Fourth, this meta-analysis cannot answer the question whether the regions of reduced GM are a consequence of alcohol consumption or whether this GM reduction is a predisposing factor for the development of AUD.

In conclusion, the findings suggest that chronic high alcohol consumption is associated with reductions in GM volume in specific brain regions. The behavioral characterization of these brain regions suggests that impairments may occur in cognitive, attentional, emotional and perceptive functions. These regions also represent nodes in behavior-relevant networks such as the salience-, frontocerebellar-, central-executive, fronto-parietal- and default-mode networks. Earlier studies have already investigated specific dysfunctions and networks, but very few studies have investigated the interaction of multiple networks in AUD. Therefore, it would be a valuable approach for future functional studies to examine the networks identified here more closely and to consider their interaction. In addition, previously unnoticed functions such as music production and comprehension as well as somesthetic functions in patients with AUD should be further explored.

## Supplementary Information

Supplementary information.
