# Supplementary material for: Meta-analysis of grey matter changes and their behavioral characterization in patients with alcohol use disorder
Source: Sci Rep. 2021 Mar 4;11:5238. doi: 10.1038/s41598-021-84804-7 (PMC7933165; doi:10.1038/s41598-021-84804-7)
Supplement: Supplementary file 1 — Supplementary information. [file 41598_2021_84804_MOESM1_ESM.pdf]

Supplementary Material

Meta-analysis of grey matter changes and their behavioral characterization in patients with Alcohol Use Disorder

Carolin Spindler[1], Sebastian Trautmann[1], Nina Alexander[1], Sonja Bröning[1,2], Sarah Bartscher[3], Markus Stuppe[4] and Markus Muehlhan[1*].

1) MSH Medical School Hamburg, Faculty of Human Sciences, Department of Psychology, Hamburg, Germany
2) MSH Medical School Hamburg, Faculty of Health Sciences, Department of Pedagogy, Hamburg, Germany
3) MSH Medical School Hamburg, Hafencity Institute for Psychotherapy, Hamburg, Germany
4) Helios Medical Center Schwerin, Carl-Friedrich-Flemming-Clinic, Department of Addiction Medicine, Schwerin, Germany

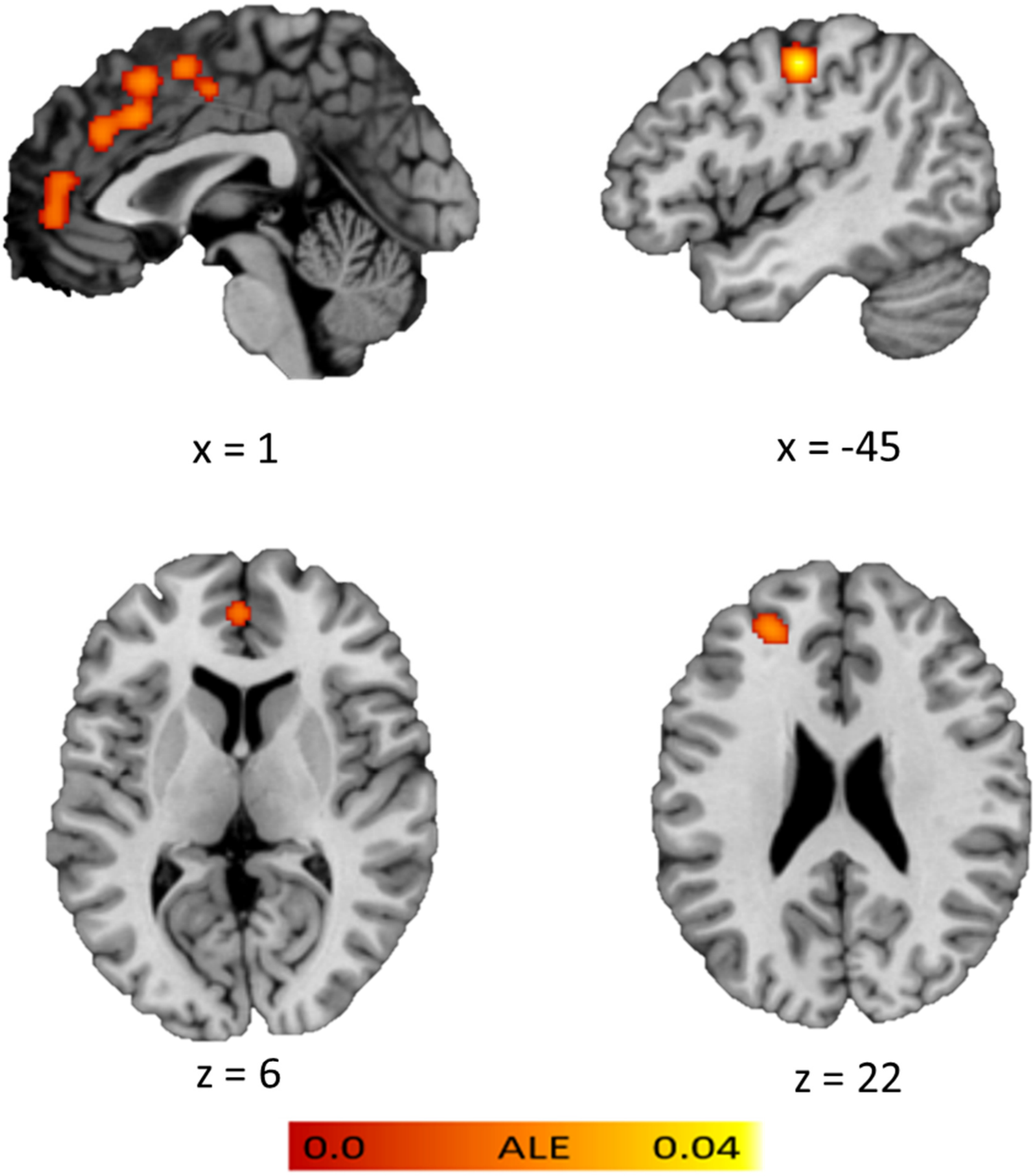

Fig. S1. Results of an ALE sub-analysis examining which regions show a change in the GM volume in the contrast AUD < HC. Cluster-forming threshold $p < 0.001$, family wise error (FWE) cluster level corrected at $p < 0.05$. x, y and z values refer to coordinates in MNI space, for detailed MNI peak voxel coordinates of the ALE clusters see table S4.

Table S1

Studies where the reported foci were pooled into one experiment.

1a	Bach et al., 2017
1b	Bach et al., 2020
2a	Chanraud et al., 2007
2b	Chanraud et al., 2009
3a	Galandra et al., 2018
3b	Galandra et al., 2020
4a	Wang et al., 2016
4b	Wang et al., 2018

References

Bach, P., Koopmann, A., Bumb, J. M., Vollstädt-Klein, S., Reinhard, I., Rietschel, M., … Kiefer, F. (2020). Leptin predicts cortical and subcortical gray matter volume recovery in alcohol dependent patients: A longitudinal structural magnetic resonance imaging study. *Hormones and Behavior*, *124*, 104749. https://doi.org/10.1016/j.yhbeh.2020.104749

Bach, P., Zois, E., Vollstädt-Klein, S., Kirsch, M., Hoffmann, S., Jorde, A., … Kiefer, F. (2017). Association of the alcohol dehydrogenase gene polymorphism rs1789891 with gray matter brain volume, alcohol consumption, alcohol craving and relapse risk. *Addiction Biology*, *24*(1), 110–120. https://doi.org/10.1111/adb.12571

Chanraud, S, Leroy, C., Martelli, C., Kostogianni, N., & Delain, F. (2009). Episodic Memory in Detoxified Alcoholics: Contribution of Grey Matter Microstructure Alteration. *PLoS ONE*, *4*(8), 6786. https://doi.org/10.1371/journal.pone.0006786

Chanraud, Sandra, Martelli, C., Delain, F., Kostogianni, N., Douaud, G., Aubin, H. J., … Martinot, J. L. (2007). Brain morphometry and cognitive performance in detoxified alcohol-dependents with preserved psychosocial functioning. *Neuropsychopharmacology*, *32*(2), 429–438. https://doi.org/10.1038/sj.npp.1301219

Galandra, C., Basso, G., Manera, M., Crespi, C., Giorgi, I., Vittadini, G., … Canessa, N. (2018). Salience network structural integrity predicts executive impairment in alcohol use disorders. *Scientific Reports*, *8*(1), 14481. https://doi.org/10.1038/s41598-018-32828-x

Galandra, C., Crespi, C., Basso, G., Manera, M. R., Giorgi, I., Poggi, P., & Canessa, N. (2020). Decreased information processing speed and decision-making performance in alcohol use disorder: combined neurostructural evidence from VBM and TBSS. *Brain Imaging and Behavior*, 1–11. https://doi.org/10.1007/s11682-019-00248-8

Wang, J., Fan, Y., Dong, Y., Ma, M., Dong, Y., Niu, Y., … Cui, C. (2018). Combining gray matter volume in the cuneus and the cuneus-prefrontal connectivity may predict early relapse in abstinent alcohol-dependent patients. *PLOS ONE*, *13*(5), e0196860. https://doi.org/10.1371/journal.pone.0196860

Wang, J., Fan, Y., Dong, Y., Ma, M., Ma, Y., Dong, Y., … Cui, C. (2016). Alterations in Brain Structure and Functional Connectivity in Alcohol Dependent Patients and Possible Association with Impulsivity. *PloS One*, *11*(8), e0161956. https://doi.org/10.1371/journal.pone.0161956

Table S2
Methodological characteristics of the studies included in the ALE meta-analysis.

#	Source	Field strength	Smooth Kernel	Software	Correction	Threshold	GM measure	Covariates	No. of Foci	Reference Space	Source of coordinates
1	Asensio et al. (2016)	1.5	8mm	SPM5	k ≥ 50	$p < 0.05$	Volume	age, total GMV and WMV	2	MNI	Table 2
2	Bach et al. (2017)	3	8mm	SPM8	k ≥ 115	$p < 0.001$	Volume	age, sex, smoking	22	MNI	Table 2
3	Bach et al. (2020)	3	8mm	SPM12	FWE and k > 10	$p < 0.05$	Volume	age, sex, abstinence, BMI, smoking and TIV	11	MNI	Table 2
4	Chanraud et al. (2007)	1.5	8mm	SPM2	FDR	$p < 0.005$	Volume	age, education, smoking	10	MNI	Table 3
5	Chanraud et al. (2009)	1.5	8mm	SPM2	FDR	$p < 0.001$	Volume	age	14	MNI	Table 3
6	Charlet et al. (2013)	3	8mm	SPM8	k ≥ 63	$p < 0.001$	Volume	age, smoking, BIS, BDI, STAI-T	36	MNI	suppl. Table 1
7	Demirakca et al. (2011)	1.5	8mm	SPM8	FWE	$p < 0.05$	Volume	age, sex, TIV	9	MNI	Table 3
8	Galandra et al. (2018)	3	8mm	SPM12	FDR	$p < 0.025$	Density	age	29	MNI	suppl. Table 2
9	Galandra et al. (2020)	3	8mm	SPM12	FWE	$p < 0.05$	Density	age, smoking	18	MNI	Table 2
10	Grodin et al. (2013)	1.5	3mm	FSL	PBNP	$p < 0.01$	Volume	age, education	18	MNI	Table 2
11	Guggenmos et al. (2017)	3	8mm	SPM12	FWE	$p < 0.05$	Volume	age, sex, site, smoking, general health	22	MNI	suppl. Table 1
12	Jang et al. (2007)	3	8mm	SPM2	FDR	$p < 0.05$	Density	-	12	TAL	Table 3
13	Mechtcheriakov et al. (2007)	1.5	10mm	SPM2	FDR	$p < 0.05$	Density	global mean voxel values, TIV	13	MNI	Table 1
14	Nurmedov et al. (2016)	1.5	8mm	SPM	FWE	$p < 0.05$	Volume	age, sex	37	MNI	Table 2
15	Pitel et al. (2012)	1.5	10mm	SPM5	FDR	$p < 0.05$	Volume	age, sex	4	MNI	p. c.
16	Rando et al. (2011)	n. a.	n. a.	SPM8	FWE	$p < 0.025$	Volume	age, iq	3	MNI	Figure 2
17	Reiter et al. (2016)	3	6mm	SPM8	FWE	$p < 0.05$	Density	TIV	10	MNI	Table 7
18	Ritz et al. (2016)	3	10mm	SPM5	FDR	$p < 0.005$	Volume	-	11	MNI	p. c.
19	Rosenthal et al. (2019)	3	8mm	SPM12	FWE and k > 30	$p < 0.005$	Volume	age, gender TIV	25	MNI	suppl. Table 2
20	Segobin et al. (2014)	1.5	8mm	SPM5	FDR	$p < 0.01$	Volume	age	4	MNI	Figure 1
21	Trick et al. (2014)	3	12mm	SPM5	FWE	$p < 0.05$	Volume	age, TIV	5	TAL	Table 4
22	Van Eijk et al. (2013)	3	8mm	SPM8	FWE	$p < 0.05$	Volume	age, sex, smoking, TIV	19	MNI	Table 3
23	van Holst et al. (2012)	3	8mm	SPM8	FDR	$p < 0.05$	Volume	age, iq, smoking, TIV	8	MNI	Table 2
24	Wang et al. (2016)	3	8mm	SPM8	FWE	$p < 0.05$	Volume	age, education, total GMV	11	MNI	Table 2
25	Wang et al. (2018)	3	8mm	SPM8	FWE	$p < 0.05$	Volume	age, education, smoking, TIV	2	MNI	Figure 1
26	Wiers et al. (2015)	3	10mm	SPM8	FWE	$p < 0.05$	Volume	age	1	MNI	Figure 2
27	Zois et al. (2017)	3	8mm	SPM8	FWE	$p < 0.05$	Volume	age, sex, BDI, smoking	20	MNI	Table 2

k=cluster size in voxels , FDR= False Discovery Rate, FWE= Family Wise Error Correction, PBNP=permutation based non-parametric testing, GMV= Gray matter volume, WMV= White matter volume, MNI= Montreal Neurological Institute, BIS= Barratt Impulsivity Scale; BDI= Beck Depression Inventory, STAI-T= Spielberger Trait-Anxiety Inventory, TIV= Total intracranial volume, SPM= Statistical Parametric Mapping, FSL= Freesurfer, BMI= Body-Mass-Index, TAL= Talairach, p.c.= personal correspondence, n.a.= information not available.

Table S3

Summarized information and allocation of the experiments contributing to the ALE clusters of convergent grey matter changes in AUD.

Source	Contributing to ALE cluster #	N AUD patients (female)	Correction method	Threshold	GM measure	No. of Foci
Asensio et al. (2016)	1	24 (0)	k ≥ 50	p < 0.05	Volume	2
Bach et al. (2017) & Bach et al. (2020)	1, 2, 3, 5, 6, 8	74 (19) 62 (14)	k ≥ 115 FWE and k > 10	p < 0.001 p < 0.05	Volume Volume	22 11
Chanraud et al. (2007) & Chanraud et al. (2009)	1	26 (0) 24 (0)	FDR FDR	p < 0.005 p < 0.001	Volume Volume	10 14
Charlet et al. (2013)	1, 2, 3, 4, 6, 7	40 (10)	k ≥ 63	p < 0.001	Volume	36
Demirakca et al. (2011)	1, 2, 7	50 (23)	FWE	p < 0.05	Volume	9
Galandra et al. (2018) & Galandra et al. (2020)	1, 2, 4, 5	23 (9) 22 (9)	FDR FWE	p < 0.025 p < 0.05	Density Density	29 18
Grodin et al. (2013)	1	37 (16)	PBNP	p < 0.01	Volume	18
Guggenmos et al. (2017)	3, 8	119 (18)	FWE	p < 0.05	Volume	22
Jang et al. (2007)	2	20 (0)	FDR	p < 0.05	Density	12
Mechtcheriakov et al. (2007)	5	22 (8)	FDR	p < 0.05	Density	13
Nurmedov et al. (2016)	2, 3, 7, 8	24 (4)	FWE	p < 0.05	Volume	37
Rando et al. (2011)	4	45 (10)	FWE	p < 0.025	Volume	3
Reiter et al. (2016)	1, 2, 4, 7	43 (9)	FWE	p < 0.05	Density	10
Ritz et al. (2016)	1	17 (4)	FDR	p < 0.005	Volume	11
Rosenthal et al. (2019)	2	46 (16)	FWE and k > 30	p < 0.005	Volume	25
Segobin et al. (2014)	4	19 (2)	FDR	p < 0.01	Volume	4
Trick et al. (2014)	1, 5	29 (11)	FWE	p < 0.05	Volume	5
Van Eijk et al. (2013)	3, 5	49 (9)	FWE	p < 0.05	Volume	19
Wang et al. (2016) & Wang et al. (2018)	1, 3, 4, 6	20 (0) 56 (0)	FWE FWE	p < 0.05 p < 0.05	Volume Volume	11 2
Zois et al. (2017)	1, 2, 3, 4, 5, 6, 7, 8	95 (24)	FWE	p < 0.05	Volume	20
**Not contributing:**						
Pitel et al. (2012)		34 (6)	FDR	p < 0.05	Volume	4
Van Holst et al. (2012)		36 (0)	FDR	p < 0.05	Volume	8
Wiers et a. (2015)		22 (0)	FWE	p < 0.05	Volume	1

FDR= False Discovery Rate, FWE= Family Wise Error Correction, PBNP=permutation based non-parametric testing.

Table S4
Subgroup Analysis of VBM studies exclusively investigating GM volume differences (referring to Table S2).
ALE clusters significant after cluster-level FWE correction for multiple comparisons.

Cluster #	Anatomical Label [a]	Peak Voxel Coordinates (MNI) x	y	z	BA	ALE (*10⁻²)[b]	Cluster Size (mm³)	Centre of mass (x, y, z)	No. of contributing experiments (%)
1	R Superior Frontal Gyrus	4	18	48	6	2.54	2104	1.9, 22.5, 38.9	10 (52.6)
	L Cingulate Gyrus	0	20	36	32	2.37			
	L Cingulate Gyrus	2	32	30	32	2.14			
2	L Postcentral Gyrus	-44	-16	48	3	4.01	1568	-45.2, -15.2, 47.2	7 (36.8)
3	L Anterior Cingulate	-2	50	2	32	1.98	1096	0.5, 48.6, 5.2	6 (31.58)
	L Anterior Cingulate	2	46	12	32	1.91			
4	L Superior Frontal Gyrus	-24	44	24	9	2.52	785	-23.8, 42.7, 24	4 (21.05)
5	L Medial Frontal Gyrus	2	2	52	6	2.06	744	0.3 ,-.4 ,50	4 (21.05)
	L Paracentral Lobe	0	-6	46	31	1.92			

BA, brodman area; L, left hemisphere; R, right hemisphere; x, y, z coordinates provided in MNI space.
a Anatomical labelling according to Talairach Daemon (nearest gray matter within 5mm, talairach.org) associated with the peak coordinates after icbm2tal transformation.
b Maximum ALE value observed in the cluster.

Table S5

Properties and FSN of the clusters resulting from the meta-analysis.

		Weighted Center (MNI)					
Cluster #	Volume (mm^3)	x	y	z	ALE ($*10^{-2}$)[a]	No. of contributing experiments (%)	FSN (%)[b]
1	3040	1.7	23.4	37.6	2.72	12(52.2)	64 (278,3)
2	1664	0.8	47.5	9.4	2.76	9 (39.1)	65 (282,6)
3	1456	-45.1	-15.1	47.2	4.01	7 (30.4)	28 (121,7)
4	1264	0.2	1.2	49.3	2.67	7 (30.4)	39 (159,6)
5	984	43.5	-14.7	8.1	2.57	6 (26.1)	5 (21,7)
6	744	-33	18.1	4	2.42	4 (17.4)	23 (100,0)
7	696	0.9	-27.2	46.8	2.12	5 (21.7)	23 (100,0)
8	680	-23.7	42.6	24.1	2.51	4 (17.4)	3 (13,0)

x, y, z coordinates provided in MNI space.
a Maximum ALE value observed in the cluster.
b Ratio to the number of experiments included in the meta-analysis.

Table S6
Single Cluster Behavioral and Paradigm Analysis.

Cluster #	Behavioral Analysis Domain (- Category)	Z-Score[a]	Paradigm Analysis Paradigm Class	Z-Score[b]
1	Action		Reward	5.87
	- Inhibition	5.55	Go/No-Go	5.54
	Cognition		Semantic Monitor/Discrimination	4.66
	- Attention	8.56	Pain Monitor/Discrimination	3.73
	- Reasoning	5.95	n-back	3.52
	- Language (Semantics)	5.67	Stroop-Color	3.50
	- Language (Speech)	4.74	Face Monitor/Discrimination	3.48
	- Memory (Working)	4.26	Cued Explicit Recognition/Recall	3.30
	- Memory (Explicit)	4.17		
	Emotion			
	- Positive (Reward/Gain)	5.81		
	- Negative (Fear)	3.80		
	Perception			
	- Somesthesis (Pain)	3.86		
2	Emotion		n. s.	
	- Positive (Reward/Gain)	3.02		
3	Action		n. s.	
	- Execution (Unspecified)	4.54		
	- Execution (Speech)	3.48		
4	Action		Pain Monitor/Discrimination	5.37
	- Execution (Unspecified)	7.09		
	Cognition			
	- Attention	3.84		
	Perception			
	- Somesthesis (Pain)	5.31		
	- Somesthesis (Unspecified)	3.99		
5	n. s.		n. s.	
6	Cognition		Reward	4.13
	- Attention	5.36	Pain Monitor/Discrimination	4.12
	- Language (Semantics)	4.79	Cued Explicit Recognition/Recall	3.51
	- Language (Speech)	4.75		
	- Memory (Explicit)	4.57		
	- Reasoning	4.37		
	- Memory (Working)	3.86		
	- Language (Phonology)	3.21		
	Emotion			
	- Positive (Reward/Gain)	4.02		
	Perception			
	- Somesthesis (Pain)	4.19		
7	n. s.		n. s.	
8	n. s.		n. s.	

a the significance threshold was set at Z-Scores ≥ 3.0 with $p \leq 0.05$ (corrected for multiple comparisons).
b the significance threshold was set at Z-Scores ≥ 3.3 with $p \leq 0.05$ (corrected for multiple comparisons).
n. s.= no results with values above significance thresholds.

Table S7a
MACM Results Cluster 1. All results were significant at a cluster-forming threshold of $p < 0.001$ and cluster level FWE corrected at $p < 0.05$.

	Peak Voxel Coordinates (MNI)					
Clusters of convergent coactivation	x	y	z	ALE-Value	Cluster Size (mm³)	No. of contributing experiments
1	-34	20	0	0.33163333	143488	383
	36	22	-2	0.3237866		
	-46	8	30	0.23798378		
	8	-16	8	0.1957702		
	48	10	30	0.1828575		
	44	38	20	0.17541309		
	-10	-16	8	0.15826043		
	-44	28	20	0.150994		
	14	8	4	0.1390841		
	-42	2	44	0.13688838		
	-30	0	52	0.11462588		
	-14	2	8	0.113632455		
	30	2	50	0.11068673		
	42	2	50	0.10641379		
	-18	6	-2	0.10494852		
	-14	8	-2	0.104593664		
	-36	52	10	0.103003874		
	30	10	50	0.097690575		
	36	58	0	0.09653247		
	10	-20	-10	0.074515685		
2	4	24	40	0.54964817	34824	422
3	-34	-52	50	0.17239326	20448	177
	-28	-60	50	0.16726567		
	-12	-68	54	0.088638134		
4	32	-60	48	0.15602237	18984	166
	42	-48	44	0.15338331		
	52	-40	44	0.12753169		
	14	-66	54	0.08704397		
	12	-70	40	0.06418817		
5	-34	-70	-24	0.08568312	4968	57
	-42	-60	-16	0.08497332		
	-48	-62	-8	0.08420824		
	-36	-58	-32	0.08041972		
	-38	-74	-14	0.058467906		
6	36	-66	-24	0.093040265	3568	43
	32	-62	-24	0.09303089		

Table S7b
MACM Results Cluster 2. All results were significant at a cluster-forming threshold of $p < 0.001$ and cluster level FWE corrected at $p < 0.05$.

	Peak Voxel Coordinates (MNI)					
Clusters of convergent coactivation	x	y	z	ALE-Value	Cluster Size (mm³)	No. of contributing experiments
1	0	54	-6	0.24690546	30856	136
	2	52	6	0.19464613		
	-8	14	-6	0.050341137		
	6	54	28	0.046710197		
	8	16	-6	0.043208823		
	-4	54	24	0.04283518		
	-10	8	0	0.039518587		
	4	32	-10	0.034798622		
	10	8	0	0.03345645		
	-8	42	34	0.026556727		
2	-2	-56	34	0.060931638	9368	43
	-6	-52	10	0.02637327		
3	-50	-64	32	0.047953356	3784	23
	-44	-72	36	0.035014596		
4	-2	-8	8	0.039817594	2072	15
	10	-16	8	0.03670676		
	-12	-12	10	0.026731731		
5	26	-8	-18	0.042187333	2032	15
6	-24	34	46	0.035304863	1808	13
	-16	36	46	0.032867692		
	-24	44	36	0.030805739		
7	-22	-8	-18	0.036467757	1352	10
	-10	-8	-12	0.029832246		
8	-60	-40	-6	0.03722415	1248	12
9	2	30	32	0.035821535	1248	11

Table S7c
MACM Results Cluster 3. All results were significant at a cluster-forming threshold of $p < 0.001$ and cluster level FWE corrected at $p < 0.05$.

	Peak Voxel Coordinates (MNI)					
Clusters of convergent coactivation	x	y	z	ALE-Value	Cluster Size (mm³)	No. of contributing experiments
1	-48	-8	50	0.33674714	67928	186
	-34	24	2	0.10316383		
	-52	12	-2	0.09674179		
	-46	4	32	0.0787183		
	-30	-56	56	0.076787524		
	-56	2	28	0.07417022		
	-26	-60	48	0.07244639		
	-54	10	22	0.06965458		
	-48	10	26	0.06799032		
	-62	-30	10	0.066048376		
	-48	-32	48	0.062875554		
	-54	-22	2	0.05832966		
	-56	-18	2	0.058148272		
	-38	-44	44	0.05572988		
	-58	-26	22	0.054233063		
	-38	-34	56	0.053416442		
	-52	-40	26	0.053196292		
	-40	-34	14	0.049284752		
	-54	-42	10	0.041775964		
2	-2	0	62	0.18160295	21272	129
	-2	6	52	0.15005694		
	6	16	42	0.09877476		
3	54	0	46	0.10635519	19552	95
	42	-4	52	0.088226184		
	50	-8	38	0.084761195		
	52	10	24	0.055808786		
	44	16	24	0.053543564		
	60	0	22	0.053188767		
	32	0	58	0.052359074		
	28	-6	50	0.040719192		
4	30	-62	-26	0.09542059	16216	71
	22	-58	-22	0.07265582		
	-42	-64	-12	0.07180755		
	-30	-62	-26	0.07052057		
	-12	-62	-18	0.05408267		
	10	-68	-18	0.052541234		
	2	-60	-18	0.052352797		
5	-12	-18	4	0.111134574	10304	65
	-22	4	4	0.082967944		

Table S7c continued

	-12	-2	14	0.040946186		
6	24	2	4	0.108101025	8168	53
	12	-18	4	0.09322881		
7	66	-32	10	0.07541736	6160	42
	58	-16	4	0.06216356		
8	36	22	2	0.09817634	5864	43
	56	10	-10	0.041534133		
9	32	-60	52	0.051839065	3472	23
	34	-46	46	0.048213158		
	48	-38	48	0.038891118		
	32	-64	40	0.03749644		

Table S7d
MACM Results Cluster 4. All results were significant at a cluster-forming threshold of $p < 0.001$ and cluster level FWE corrected at $p < 0.05$.

	Peak Voxel Coordinates (MNI)					
Clusters of convergent coactivation	x	y	z	ALE-Value	Cluster Size (mm³)	No. of contributing experiments
1	0	10	50	0.69207853	236664	400
	-32	20	2	0.25548097		
	36	20	0	0.2549885		
	-12	-16	8	0.23443471		
	-46	6	30	0.2139869		
	10	-16	8	0.20276067		
	50	10	28	0.20274189		
	-46	-2	44	0.19726895		
	-26	-62	50	0.1753548		
	24	2	4	0.16268954		
	48	16	-4	0.15998583		
	-40	-16	54	0.1598747		
	-20	6	2	0.15770315		
	-26	-4	54	0.15695606		
	34	-2	56	0.1471871		
	36	-50	48	0.14329492		
	40	-2	52	0.14191836		
	-50	8	2	0.13978432		
	28	-64	54	0.1286012		
	38	44	28	0.10803274		
	62	-32	28	0.10365503		
	-60	-26	8	0.08832339		
	48	-36	46	0.08773879		
	-52	-24	20	0.08732655		
	-38	44	24	0.08301589		
	38	54	10	0.07572825		
	54	-26	42	0.06816243		
	-26	-76	32	0.06708826		
	32	-72	32	0.06550684		
	-50	-34	12	0.06373383		
	-60	-10	4	0.059944507		
2	-34	-56	-30	0.11084647	10416	106
	-42	-60	-16	0.10423207		
	-26	-64	-24	0.09039151		
	-38	-74	-12	0.07887538		
3	32	-62	-26	0.1176925	5664	69
	44	-60	-18	0.06764966		
4	60	-24	8	0.09303442	3528	40

Table S7e
MACM Results Cluster 5. All results were significant at a cluster-forming threshold of *p* < 0.001 and cluster level FWE corrected at *p* < 0.05.

	Peak Voxel Coordinates (MNI)					
Clusters of convergent coactivation	x	y	z	ALE-Value	Cluster Size (mm³)	No. of contributing experiments
1	-46	-22	6	0.075857356	22656	60
	-50	-16	2	0.071482964		
	-56	-12	12	0.04910549		
	-46	8	-2	0.042659517		
	-58	-4	20	0.039180387		
	-62	-26	22	0.03293335		
	-52	10	-4	0.03235797		
	-36	20	2	0.031398352		
2	50	-12	4	0.19495137	18832	82
	62	-24	22	0.036936358		
	60	0	18	0.03622244		
	48	-10	38	0.034469627		
3	-4	-2	62	0.049664114	4904	22
	6	16	36	0.03918255		
	0	4	50	0.037966803		
4	44	8	4	0.043459233	3800	18
	36	20	4	0.037898652		
5	-50	-8	46	0.045419127	3056	18
6	-24	-62	-20	0.041024506	2360	14
	-18	-62	-20	0.040627137		
7	-8	-14	4	0.03542749	2128	14
	-12	-28	2	0.02441962		
8	24	2	6	0.04158319	2112	13
	26	0	-6	0.032958817		
9	-26	-6	-6	0.041825753	1832	14
	-22	-4	-18	0.024585413		
10	12	-16	6	0.037333004	1744	10
11	18	-60	-20	0.043022238	1440	11

Table S7f
MACM Results Cluster 6. All results were significant at a cluster-forming threshold of $p < 0.001$ and cluster level FWE corrected at $p < 0.05$.

	Peak Voxel Coordinates (MNI)					
Clusters of convergent coactivation	x	y	z	ALE-Value	Cluster Size (mm³)	No. of contributing experiments
1	-34	20	0	0.6778235	132136	303
	36	22	-2	0.37349984		
	-46	6	30	0.21459693		
	48	8	26	0.17455442		
	-10	-14	4	0.1555588		
	14	6	0	0.14325024		
	10	-16	6	0.14198972		
	-54	10	8	0.12271503		
	-46	26	22	0.12187719		
	-20	8	2	0.109429464		
	48	4	42	0.10425648		
	44	38	20	0.09739923		
	-28	0	54	0.09293294		
	44	28	26	0.084873214		
	-38	46	20	0.08185789		
	28	2	52	0.0795518		
	-44	-4	52	0.079535425		
	20	-4	-12	0.077424705		
	-36	-4	58	0.074888684		
	40	48	10	0.07260532		
	-6	-20	-12	0.07140302		
	44	50	-2	0.06908849		
	-20	-4	-14	0.06472745		
	-38	52	6	0.06365843		
	8	-20	-12	0.056404267		
2	0	16	46	0.22630031	28752	233
	4	20	42	0.22422123		
	-2	34	26	0.08522093		
3	32	-56	50	0.10961987	17496	131
	44	-46	46	0.102738276		
	60	-28	6	0.08526672		
	62	-42	32	0.07413719		
	56	-24	20	0.06651594		
	60	-32	18	0.06592737		
	14	-68	54	0.06212902		
	54	-44	16	0.059666086		
4	-32	-52	46	0.14485683	17032	137
	-26	-66	48	0.13219751		
	-46	-42	48	0.11258497		
	-46	-38	46	0.112434946		

Table S7f continued

	-42	-44	44	0.11013844		
5	-44	-60	-14	0.10913099	11088	93
	-36	-56	-30	0.08374652		
	-30	-64	-28	0.082052335		
6	32	-64	-26	0.09590842	3120	40
	46	-64	-4	0.05967176		

Table S7g
MACM Results Cluster 7. All results were significant at a cluster-forming threshold of $p < 0.001$ and cluster level FWE corrected at $p < 0.05$.

	Peak Voxel Coordinates (MNI)					
Clusters of convergent coactivation	x	y	z	ALE-Value	Cluster Size (mm³)	No. of contributing experiments
1	4	-16	50	0.07811668	8432	32
	2	-26	50	0.06981361		
	6	-8	66	0.015520015		
	0	-4	42	0.013998251		
2	36	18	4	0.025004093	1592	8
	44	10	2	0.02043405		
	58	14	2	0.013594652		

Table S7h
MACM Results Cluster 8. All results were significant at a cluster-forming threshold of $p < 0.001$ and cluster level FWE corrected at $p < 0.05$.

	Peak Voxel Coordinates (MNI)					
Clusters of convergent coactivation	x	y	z	ALE-Value	Cluster Size (mm³)	No. of contributing experiments
1	8	26	30	0.030556392	6560	19
	-4	10	52	0.030547412		
	6	16	46	0.019976819		
	8	10	62	0.019708596		
	-4	14	42	0.019433334		
2	-26	50	18	0.13117135	5952	35
3	-32	22	4	0.03542612	2616	13
	-36	10	8	0.019815054		
4	42	18	2	0.023411926	2424	9
	34	24	6	0.017192915		

Table S8

Study inclusion and exclusion differences between previous and current meta-analysis (MA).

**Included in current MA**	**Xiao et al. (2015)**	**Yang et al. (2016)**	**Klaming et al. (2019)**
Asensio et al. (2016)			
Bach et al. (2017)			
Bach et al. (2020)			
Chanraud et al. (2007)	X	X	X
Chanraud et al. (2009)		X	
Charlet et al. (2014)	X		X
Demirakca et al. (2011)	X	X	X
Galandra et al. (2018)			
Galandra et al. (2020)			
Grodin et al. (2013)	X	X	X
Guggenmos et al. (2017)			
Jang et al. (2007)	X	X	X
Mechtcheriakov et al. (2007)	X	X	X
Nurmedov et al. (2016)			X
Pitel et al. (2012)			
Rando et al. (2011)	X		X
Reiter et al. (2016)			
Ritz et al. (2016)			
Rosenthal et al. (2019)			
Segobin et al. (2014)		X	X
Trick et al. (2014)			
Van Eijk et al. (2013)			
van Holst et al. (2012)	X	X	X
Wang et al. (2016)			X
Wang et al. (2018)			
Wiers et al. (2015)			X
Zois et al. (2017)			X
**Excluded in current MA (reasons)**			
Brooks et al., 2014 (adolescent sample)		X	
Dalvie et al., 2014 (adolescent sample)		X	
Fein et al. 2013, (adolescent sample)		X	
Howell et al.,2013 (adolescent sample)		X	
Li et al., 2011 (no access, chinese language)	X		

Table S9

Checklist for neuroimaging meta-analysis according to Müller et al. (2018).

The research question is specifically defined	YES, and it includes the following contrast:<br><br><u>whole brain GM changes in AUD vs whole brain GM changes in HCs.</u><br><br>***→ Introduction, last paragraph***
The literature search was systematic	YES, it included the following keywords in the following databases:<br><br><u>Keywords</u>: (Alcohol Dependence OR Dependence, Alcohol OR Alcohol Addiction OR Addiction, Alcohol OR Alcoholic Intoxication, Chronic OR Chronic Alcoholic Intoxication OR Intoxication, Chronic Alcoholic OR Alcohol Use Disorder OR Alcohol Use Disorders OR Use Disorder, Alcohol OR Use Disorders, Alcohol OR Alcohol Abuse OR Abuse, Alcohol) AND (voxel-based morphometry OR VBM OR structural MRI).<br><br><u>Databases:</u> PubMed, PsycINFO and Web of Science databases (up to June 1, 2020).<br><br>***→ Methods section: Literature Search, Study Selection and Data Extraction***
Detailed inclusion an exclusion criteria are included	YES, and reasons of non-standard criterion were:<br><br><u>Exclusion of studies reporting null-findings - they can not be taken into account because they do not provide spatial coordinates, which are a prerequisite for the coordinate-based meta-analytical approach.</u><br><br>***→ Methods section: Literature Search, Study Selection and Data Extraction***
Sample overlap was taken into account	YES, using the following method:<br><br><u>Pooling the foci into one experiment and considering the smaller sample size in a conservative approach.</u><br><br>***→ Methods section: Literature Search, Study Selection and Data Extraction***
All experiments use the same search coverage (state how brain coverage is	YES, the search coverage is the following:

assessed and how small volume corrections and conjunctions are taken into account)	whole brain coverage only, verified via details of the scanner parameters provided in the method section of the papers and average brain sizes provided by Müller et al. 2018.<br><br>→ ***Methods section: Literature Search, Study Selection and Data Extraction***
Studies are converted to a common reference space	Yes, using the following conversion:<br><br>Coordinates reported in Talairach space were transformed into MNI space using the Lancaster transform icbm2tal implemented in GingerALE.<br><br>→ ***Methods section: Anatomical Likelihood Estimation***
Data extraction have been conducted by two investigators (ideal case) or double checked by the same investigator (state how double-checking was performed)	YES, the following authors:<br><br>CS checked the inclusion criteria<br>CS extracted coordinates<br>CS extracted other info: demographic sample characteristics and methodological characteristics<br>MM double-checked for the following data: all<br><br>→ ***Methods section: Literature Search, Study Selection and Data Extraction***
The paper includes a table with at least the references, basic study description (e. g. for fMRI tasks: stimuli), contrasts and basic sample descriptions (e.g. size, mean age and gender distribution, specific characteristics) of the included studies, source of information (e.g. contact with authors), reference space	YES, and also the following data:<br><br>MRI: field strength<br>Pre-processing: Smooth Kernel, Software<br>Analysis: Method and threshold of correcting for multiple comparisons, GM Measure, Covariates<br>Results: No. Foci, Reference Space, Source of coordinates<br><br>Clinical sample characteristics: Diagnosis and Duration, Abstinence Duration<br><br>→ ***Table 1 and table S2***
The study protocol was previously registered and all analyses planned beforehand, including the methods and parameters used for inference, correction for multiple testing, etc.	Yes:<br>1) The meta-analysis was registered before starting the starting the search at:<br>PROSPERO (CRD42020190710)<br><br>→ ***Methods section: Beginning***<br><br>2) Any non-planned analyses are clearly stated as post-hoc or non-prespecified in the paper.<br>1. Sub-analysis of 'volume' studies<br>2. Assessment of Publication Bias

	→ ***Methods section, Results and Discussion***<br><br>3) The meta-analysis used the default methods and parameters of the software.<br><br>→ ***Methods section: Anatomical Likelihood Estimation***
The meta-analysis includes diagnostics	YES, the following:<br><br><u>Number of experiments contributing to the resulting ALE-clusters and summarized information and allocation of the experiments contributing to the ALE clusters of convergent grey matter changes in AUD.</u><br><br>→ ***Table 2 and table S3***
